# Autoinducer-2 signaling promotes intestinal colonization of *Aeromonas veronii* and induces cell apoptosis in loach (*Misgurnus anguillicaudatus*)

**DOI:** 10.1128/aem.00143-25

**Published:** 2025-02-13

**Authors:** Yi Li, Shuo Han, Wenfang Niu, Chao Gao, Yuqi Wang, Mengyuan Qin, Jingjing Han, Xiaohua Xia, Hailei Wang

**Affiliations:** 1 College of Life Sciences, Henan Normal University 66519 , Xinxiang, China; 2 Henan Province Engineering Laboratory for Bioconversion Technology of Functional Microbes, Xinxiang, China; 3 School of Medicine, Qingdao Huanghai University 563427 , Qingdao, China; Universita degli Studi di Napoli Federico II, Portici, Italy

**Keywords:** *Aeromonas veronii *Z12, autoinducer-2, intestinal colonization, reactive oxygen species, p53-mediated cell apoptosis

## Abstract

Quorum sensing systems, particularly autoinducer-2 (AI-2) signaling, have significant effects on bacterial colonization and virulence. However, how they affect intestinal colonization by pathogens and subsequent host immune responses remains unclear. Here, we investigated the influence of AI-2 signaling on the intestinal colonization ability of *Aeromonas veronii* Z12 and the host’s immune response. We found that AI-2 signaling promoted the colonization of *A. veronii* to the intestine of loach (*Misgurnus anguillicaudatus*) and caused severe intestinal damage, while D-ribose, an AI-2 signaling inhibitor, effectively inhibited the colonization of *A. veronii*. Transcriptomic sequencing elucidated the molecular mechanism of this damage, revealing upregulation of p53 pathway genes associated with apoptosis. Furthermore, intestinal microbiota dysbiosis induced by *A. veronii* colonization was associated with host cell apoptosis, leading to nitrite accumulation, which increased intracellular reactive oxygen species (ROS) levels, which activated the p53 pathway, and induction of cell apoptosis. These findings provide insights into the interaction among bacterial quorum sensing, intestinal microbiota, and the host immune response, which highlight potential therapeutic targets for mitigating bacterial-induced intestinal damage.

**IMPORTANCE:**

The intestinal colonization of pathogens regulated by autoinducer-2 (AI-2) signaling and its induced host response have not been fully characterized. Here, we revealed the effect of AI-2 on intestinal colonization of *Aeromonas veronii* and its induced cell apoptosis in loach. Our study demonstrated that the deficiency of AI-2 significantly reduced *A. veronii* colonization in the loach intestine and mitigated the tissue damage. Additionally, *A. veronii* colonization induced significant upregulation of p53 pathway genes and proteins, indicating a key role of AI-2 signaling in host responses. Understanding these mechanisms not only helps to elucidate the pathogenicity of *A. veronii* but also may provide broader insights into the pathogenic mechanisms of other pathogens, thus revealing general principles of pathogen–host interactions across different models. Furthermore, we found that *A. veronii* colonization led to intestinal microbiota dysbiosis, notably an increase in the abundance of *Hypomicrobium* sp., which was associated with nitrite accumulation, elevating reactive oxygen species levels, activating the p53 pathway, and inducing cell apoptosis. These findings provide important insights into the complex mechanisms of AI-2 signaling in bacterial–host interactions. Additionally, the regulatory role of AI-2 signaling may have potential clinical applications as an intervention strategy, offering new directions for developing treatments against intestinal infections.

## INTRODUCTION

The intestine serves as a vital organ in aquatic animals, where its stable microbial community structure plays a crucial protective role in the host’ s health (1). Intestinal microbiota participates in numerous processes, including nutrient absorption, immune regulation, and the resistance to pathogens (2). However, invasion by bacterial pathogens can induce intestinal microbiota dysbiosis, leading to the excess proliferation of pathogens, thereby posing a severe threat to host health. *Aeromonas veronii*, a common zoonotic pathogen, belongs to the phylum Proteobacteria, class Gammaproteobacteria, and family Aeromonadaceae. This gram-negative bacterium typically exhibits a rod shape and motility that enhance its ability to colonize host environments (3). *A. veronii* infection can cause a serious infectious disease called the motile Aeromonas *enteritis*, which will inflict severe damage on health of aquatic animals and humans, especially to children or human with low immunity (4). In the intestinal environment, *A. veronii* may colonize through multiple strategies such as adhesion, biofilm formation, and secretion of virulence factors, interacting with the intestinal microbial community and the host immune system (5). Furthermore, apoptosis is a crucial biological process in the intestine, which contributed to maintaining cellular balance and tissue repair (6). However, pathogens, such as *A. veronii*, may disrupt this process, leading to imbalances that damage intestinal tissue structure and increase the risk of intestinal diseases. The regulatory role of the interspecies signal autoinducer-2 (AI-2) in pathogenic bacteria, particularly its influence on colonization dynamics and host responses, remains a topic of interest.

Previous studies have addressed that many physiological behaviors of *A. veronii* are regulated by quorum sensing systems, including the AhyRI system mediated by N-acyl-homoserine lactones (AHLs) signals, the LuxS/AI-2 system mediated by AI-2 signals, and the QseBC system mediated by autoinducer-3 (5). Moreover, the interspecies AI-2 signaling in microbial communities has garnered attention for its regulatory role in pathogenic bacteria. AI-2 signaling not only directly regulates physiological processes such as biofilm formation, adhesion, and secretion of virulence factors but also indirectly modulates pathogenicity through interactions within microbial communities and host–microbe interactions (7). Additionally, recent studies have demonstrated that AI-2 can interact directly with host cell proteins, influencing immune responses and inflammatory pathways. For example, AI-2 has been shown to activate the NF-κB signaling pathway in host cells, promoting inflammation and immune responses (8). Furthermore, regulation of host on quorum sensing molecules like AI-2 is essential for understanding bacterial pathogenicity (9). These findings suggest that the role of AI-2 extends beyond bacterial communication, impacting host–pathogen interactions. In hosts, AI-2 signaling may alter microbial community structure and composition, thereby influencing host immune responses. Our previous studies have demonstrated that AHLs signals can promote *A. veronii* colonization in zebrafish intestines, leading to intestinal microbiota dysbiosis and intestinal damage (10). Furthermore, AI-2 signals can modulate *A. veronii* adhesion to blood cells of loach through participation by c-di-GMP (5). However, whether AI-2 signals mediate *A. veronii* colonization in the host intestine, causing intestinal microbiota dysbiosis and subsequent host immune response, remains incompletely defined.

Therefore, this study aimed to explore AI-2 signal-mediated *A. veronii* intestinal colonization and its effects on intestinal microbial community and host immune responses. The results indicate that AI-2 promotes *A. veronii* colonization, leading to alterations in intestinal microbial community, accumulation of nitrites, and activation of p53 apoptosis pathway in the host intestines, ultimately causing severe intestinal damage. This finding expands our understanding of AI-2 signal-mediated regulation of pathogen colonization and delves into host immune and intestinal microbial responses, providing new insights into the role of quorum sensing signals in microbial colonization and host interaction.

## RESULTS

### The deficiency of AI-2 signaling leads to a decrease in the adhesion and colonization ability of *A. veronii*

**Fig 1:**
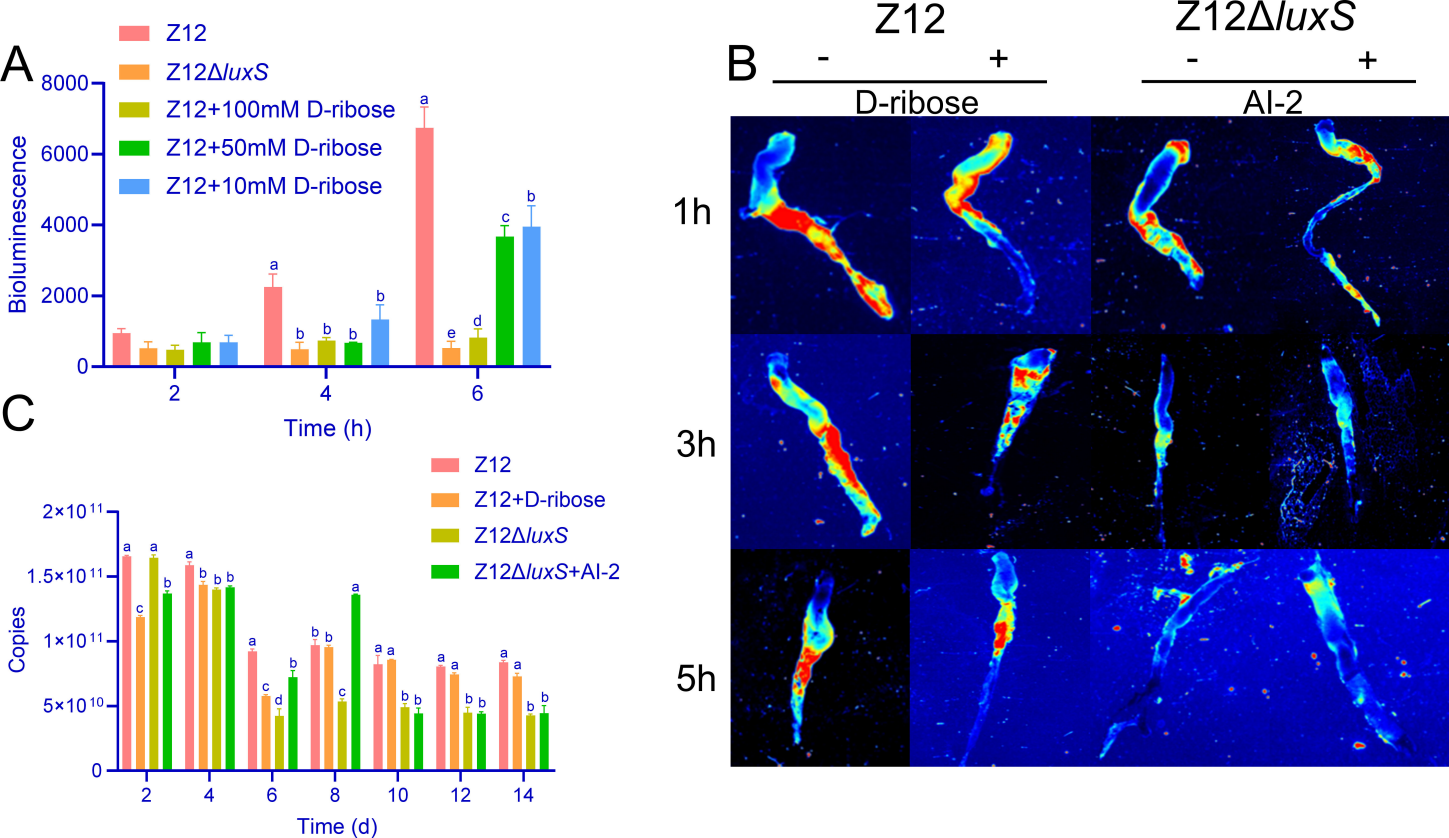
Inhibitory effect of D-ribose on AI-2 signaling and colonization of different strains and treatments in the intestine. (**A**) Bioluminescence of *V. harveyi* in the presence of cell-free supernatant of strain Z12, Z12Δ*luxS*, and Z12 with the addition of different concentrations of D-ribose. (**B**) The adhesion ability of FITC-labeled Z12 and Z12Δ*luxS* to the loach intestine with or without exogenous D-ribose and AI-2. The bacterial cells were labeled with FITC before intestinal administration. After dissection, the colonization level was visualized under fluorescence microscopy. The red fluorescence intensity represented bacterial abundance. (**C**) Colonization of different *A. veronii* strains in the loach intestines with or without adding exogenous D-ribose and AI-2. Means of groups not sharing a common letter are significantly different at *P* < 0.05, as determined by a ANOVA. The statistical significance threshold was set at *P* < 0.05 for all comparisons.

To investigate whether AI-2 signaling affects the adhesion and colonization ability of *A. veronii* Z12 to the intestine, we determined the adhesion and colonization ability of *A. veronii* Z12Δ*luxS*, a *luxS* gene deletion mutant that lost the ability to synthesize AI-2. Furthermore, D-ribose, a reported AI-2 antagonist, was used to verify interference with the AI-2 signaling produced by strain Z12 using a reporter strain luminescence assay, where the luminescence level of the reporter strain *Vibrio harveyi* BB170 reflected the intensity of AI-2 signaling in the supernatant. As shown in Fig. 1A, the bioluminescence of *V. harveyi* BB170 with the addition of strain Z12 supernatant reached its highest level at 6 h, indicating the highest AI-2 content at this time, while the bioluminescence of *V. harveyi* BB170 with the addition of Z12Δ*luxS* supernatant remained at a lower level and did not increase with time. However, the addition of D-ribose to the supernatant of strain Z12 significantly reduced the bioluminescence of *V. harveyi* BB170, with the most pronounced effect observed after adding 100 mM of D-ribose, suggesting that D-ribose effectively blocked the action of AI-2 signaling. Subsequently, we assessed the adhesion ability of different *A. veronii* strains to the intestine. As shown in Fig. 1B, the fluorescence intensity produced by fluorescein 5-isothiocyanate (FITC)-labeled bacteria in the intestines of the Z12 group was significantly higher than that in the Z12Δ*luxS* group. However, the addition of D-ribose could effectively interfere AI-2 signaling, thus reducing the adhesion of strain Z12 to the intestine of loach, which resulted in a decreasing fluorescence intensity. This effect was most pronounced at 3 h, while adding AI-2 to the Z12Δ*luxS* group effectively restored its adhesion ability, indicating that AI-2 signaling effectively promotes the adhesion of strain Z12 to the intestine.

Furthermore, intestinal colonization experiments showed that the number of each bacterial strain colonizing the intestine decreased initially and then stabilized over time. Except for the second day, the number of bacterial cells of strain Z12 in the intestine was significantly higher than that of strain Z12Δ*luxS*. During the early stages of the experiment, D-ribose significantly inhibited the colonization of strain Z12 in the intestine. However, this inhibitory effect gradually weakened with the process time, disappearing completely after the 8th day. Similarly, the exogenous AI-2 could enhance the colonization ability of strain Z12Δ*luxS* in the intestine during the early stages of the experiment, but this promoting effect diminished over time, disappearing completely by the 10th day. After the 10th day, the bacterial cells of strain Z12 in the intestine were always significantly higher than that of strain Z12Δ*luxS* (Fig. 1C). Accordingly, these results indicate that AI-2 signaling promotes the adhesion and colonization of strain Z12 to the intestine, and D-ribose can block the adhesion and colonization of strain Z12 by interfering with AI-2 signaling.

### The colonization mediated by AI-2 signaling of *A. veronii* leads to intestinal damage

**Fig 2:**
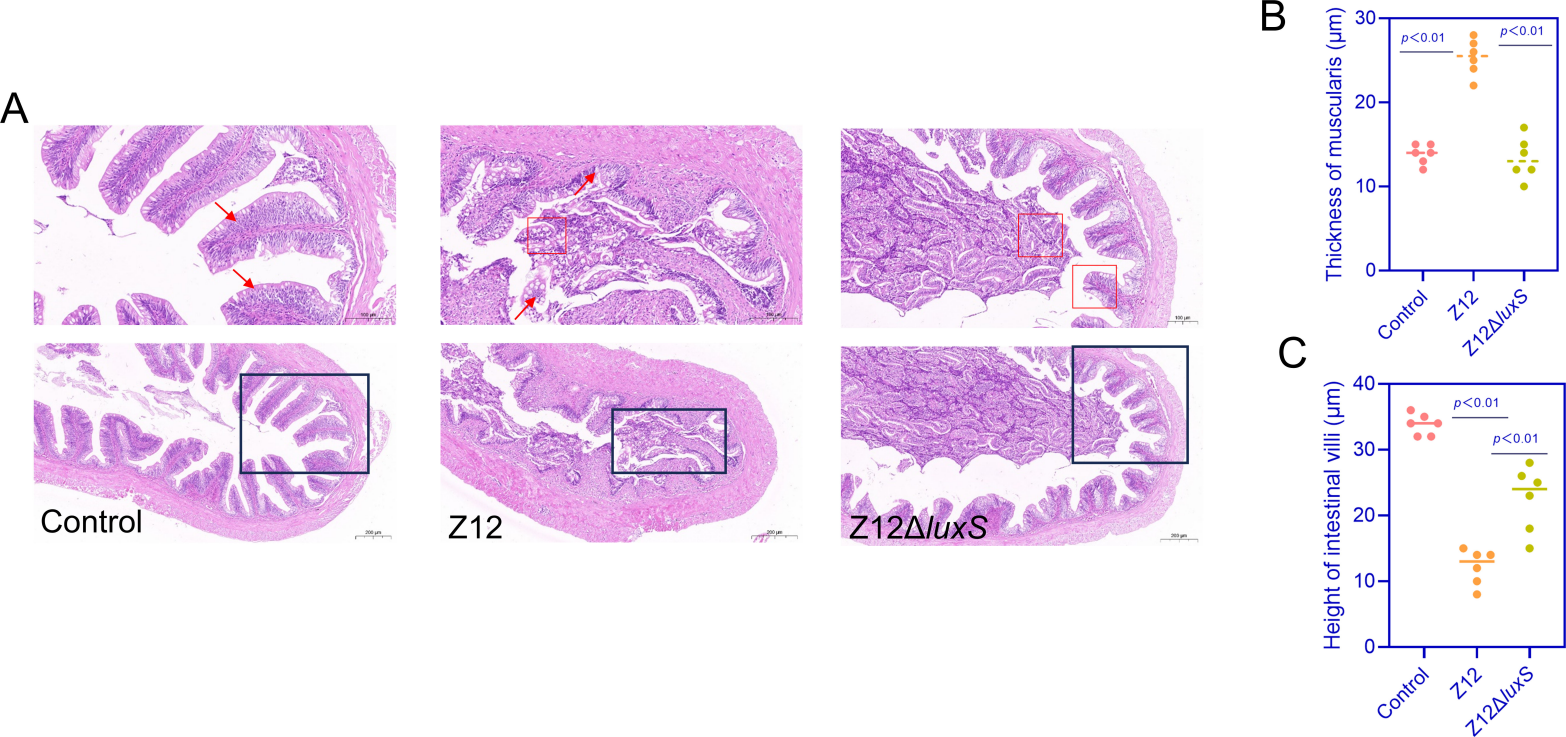
Histopathological analysis of loach intestines infected by *A. veronii*. (**A**) H&E staining of loach intestines, with red arrows indicating damaged structures. (**B**) Thickness of muscularis. (**C**) Height of intestinal villi. Red boxes indicate the area of obvious cell damage in the tissue section, and black boxes represent the same area after magnification. Scale bars represent 200 µm.

Histopathological analysis of intestinal tissues revealed that colonization of strain Z12 in the intestine caused severe tissue damage. As shown in Fig. 2A, the morphology of intestinal tissues in the control group was intact, with normal cellular arrangement, dense and orderly. There was no apparent damage to the thickness of the intestinal muscle layer and the length of intestinal villi. In contrast, the intestinal tissues in the Z12 group exhibited severe damage, with thinning of the intestinal wall, cellular injury, and deformation, blurred cell nuclei with nuclear pyknosis, slight detachment of circular and longitudinal muscle layers, occasional separation of epithelial cells from the lamina propria, and significant shortening or even rupture of intestinal villi. The intestinal structure integrity in the Z12Δ*luxS* group was significantly better than that in the Z12 group, with most intestinal villi maintaining intact structure and morphology, significantly higher villus height compared to the Z12 group, minimal shedding of intestinal tissues, and a noticeable increase in intestinal wall thickness compared to the Z12 group (Fig. 2B and C). These results indicate that AI-2 promotes the colonization of *A. veronii* and further causes damage to intestinal tissues.

### Transcriptomic sequencing reveals the molecular mechanism of *A. veronii* colonization mediated by AI-2 signaling, and its impact on intestinal damage

**Fig 3:**
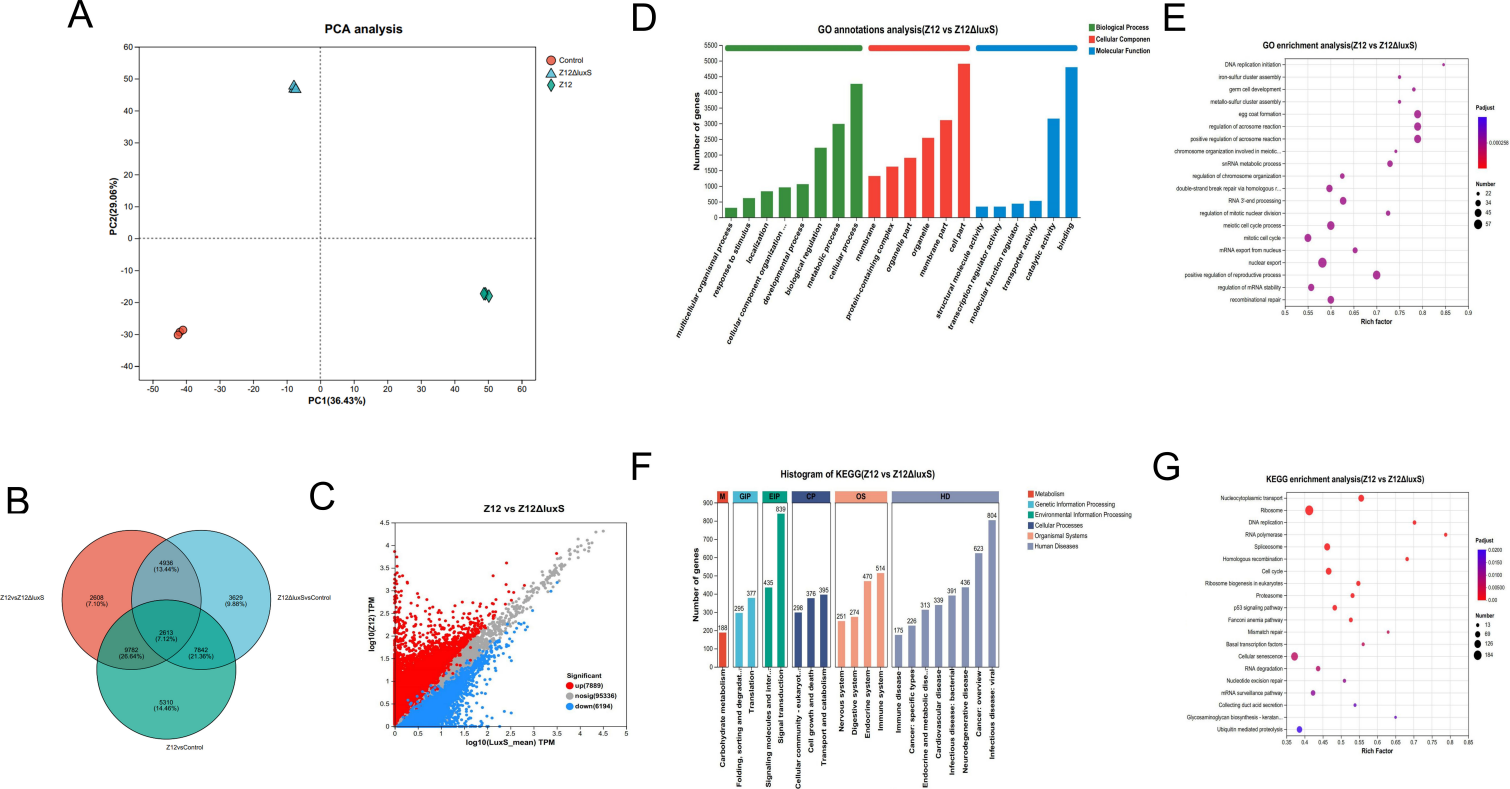
Transcriptome sequencing of loach intestines after infected by different *A. veronii* strains. (**A**) PCA of the transcriptomes of each sample. The plot shows the distribution of different samples based on the first two principal components (PC1 and PC2), which explain 36.43% and 29.06% of the total variance, respectively. (**B**) Venn diagrams between treatments. (**C**) Scatterplot of DEGs between Z12 and Z12Δ*luxS*. (**D, E**) Bar and bubble charts of GO enrichment for DEGs between Z12 and Z12Δ*luxS*. (**F, G**) Bar and bubble charts of the KEGG pathway enrichment for differential genes between Z12 and Z12Δ*luxS*.

To further elucidate the molecular mechanism of intestinal damage caused by *A. veronii* colonization, we performed intestinal transcriptome sequencing analysis. Principal component analysis (PCA) revealed substantial distances between the sample points of different treatments, with clear separation among the three samples within each treatment group, indicating significant differences in gene expression among the three treatment groups, while biological replicates within each treatment group were closely clustered (Fig. 3A). Differential gene expression between different treatment groups was depicted in Fig. 3B, showing that compared to the Control group, the Z12 and Z12Δ*luxS* groups induced significant differential expression in 5310 and 3629 genes, respectively, accounting for 14.46% and 9.88% of the total differentially expressed genes (DEGs), while 2,608 genes exhibited significant differential expression between Z12 and Z12Δ*luxS*, representing 7.10% of the total. Further comparisons among treatments yielded scatterplots of DEGs, revealing that compared to the Z12Δ*luxS* group, 7,889 genes and 6,194 genes were upregulated and downregulated in the Z12 group, respectively. (Fig. 3C).

Gene Ontology (GO) annotation revealed that the dominant GO categories for DEGs between Z12 and Z12Δ*luxS* were Cellular Process, Metabolic Process, and Biological Regulation. While in the Kyoto Encyclopedia of Genes and Genomes (KEGG) pathways, Carbohydrate Metabolism, Translation, Signal Transduction, Immune System, and Infectious Disease were predominant (Fig. 3D and E).

Subsequent enrichment analysis of differential genes revealed that the top 20 pathways significantly enriched between Z12 and Z12Δ*luxS* groups included DNA Replication Initiation, Iron-Sulfur Cluster Assembly, Germ Cell Development, Metallo-Sulfur Cluster Assembly, and Egg Coat Formation (Fig. 3F). In KEGG enrichment analysis results, DEGs were mainly enriched in Nucleocytoplasmic Transport, Ribosome, DNA Replication, RNA Polymerase, and Spliceosome pathways (Fig. 3G). Of note, in KEGG enrichment analysis, we found that compared to the Z12Δ*luxS* group, the expression of genes related to the p53 signaling pathway associated with apoptosis was significantly upregulated in the Z12 group, indicating activation of the apoptotic pathway in host cells following infection by wild-type strain Z12.

### *A. veronii* colonization mediated by AI-2 activates the host intestinal cell p53 apoptosis pathway

**Fig 4:**
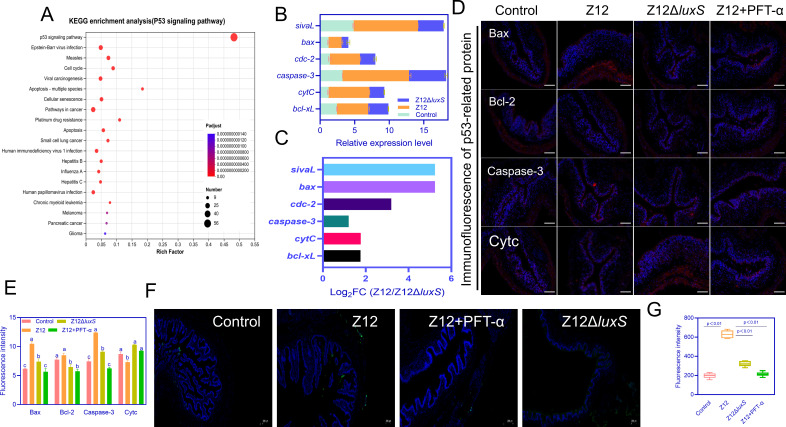
Cell apoptosis mediated by the p53 pathway in the intestines infected by *A. veronii*. (**A**) KEGG enrichment of genes related to the p53 pathway. (**B**) Quantitative PCR validation of p53 pathway-related genes. (**C**) Transcriptome sequencing results of p53 pathway-related genes. (**D**) Immunofluorescence validation of p53-related protein expression. Scale bars represent 200 µm. (**E**) Fluorescence values of immunofluorescence histochemistry. (**F**) TUNEL staining of loach intestines. Scale bars represent 200 µm. (**G**) Fluorescence values of TUNEL assay. Means of groups not sharing a common letter are significantly different at *P* < 0.05.

Enrichment analysis of the p53 pathway using KEGG revealed that in the Z12 group, pathways indirectly regulated by p53, such as Epstein–Barr virus infection, Viral carcinogenesis, and Apoptosis-multiple species, were enriched (Fig. 4A). Among these, the p53 pathway with the highest enrichment comprised 56 DEGs. Specifically, in the Z12 group, 38 genes directly associated with apoptosis were upregulated, including *caspase-3*, *cytC*, *bid*, *bax*, *bcl-xL*, and *sivaL*. Subsequently, quantitative PCR validation of these genes was performed, which showed significantly higher expression levels in the Z12 group compared to the Control, with *caspase3* exhibiting the highest upregulation followed by *sivaL* (Fig. 4B). Moreover, the expression levels of these genes were downregulated in the Z12Δ*luxS* group compared to the Z12 group, approaching levels similar to the control group. This result corroborates the trend observed in transcriptomic sequencing (Fig. 4C), further validating its reliability.

Immunofluorescence analysis also confirmed this trend, with Caspase-3 expression being highest in the Z12 group, followed by the Z12Δ*luxS* group. However, the anti-apoptotic Bcl-2 protein showed significantly lower expression in Z12 compared to the Control group, indicating significant inhibition of the anti-apoptotic pathway in the host intestine following strain Z12 infection. Additionally, the pro-apoptotic factor Bax exhibited significantly higher fluorescence intensity in the Z12 group compared to the other three treatment groups, while cytochrome CytC fluorescence intensity did not show significant differences among the treatment groups (Fig. 4D and E). These results suggest that strain Z12 activates key factors promoting apoptosis within the p53 pathway, such as Bax and Caspase-3 expression, leading to cell apoptosis upon colonizing the host intestine by strain Z12. Subsequent terminal deoxynucleotidyl transferase dUTP nick end labeling (TUNEL) revealed that cells marked by SpGreen were significantly more abundant in the Z12 group compared to other groups, indicating the most severe apoptosis in this group, followed by the Z12 group after treatment with pifithrin-α, a specific inhibitor of p53, which notably suppressed cell apoptosis (Fig. 4F and G). Altogether, it can be inferred that strain Z12 colonization induces p53-mediated cell apoptosis, thus confirming the findings of the previous transcriptomic sequencing.

### *A. veronii* colonization disrupts the structure of the host intestinal microbiota

**Fig 5:**
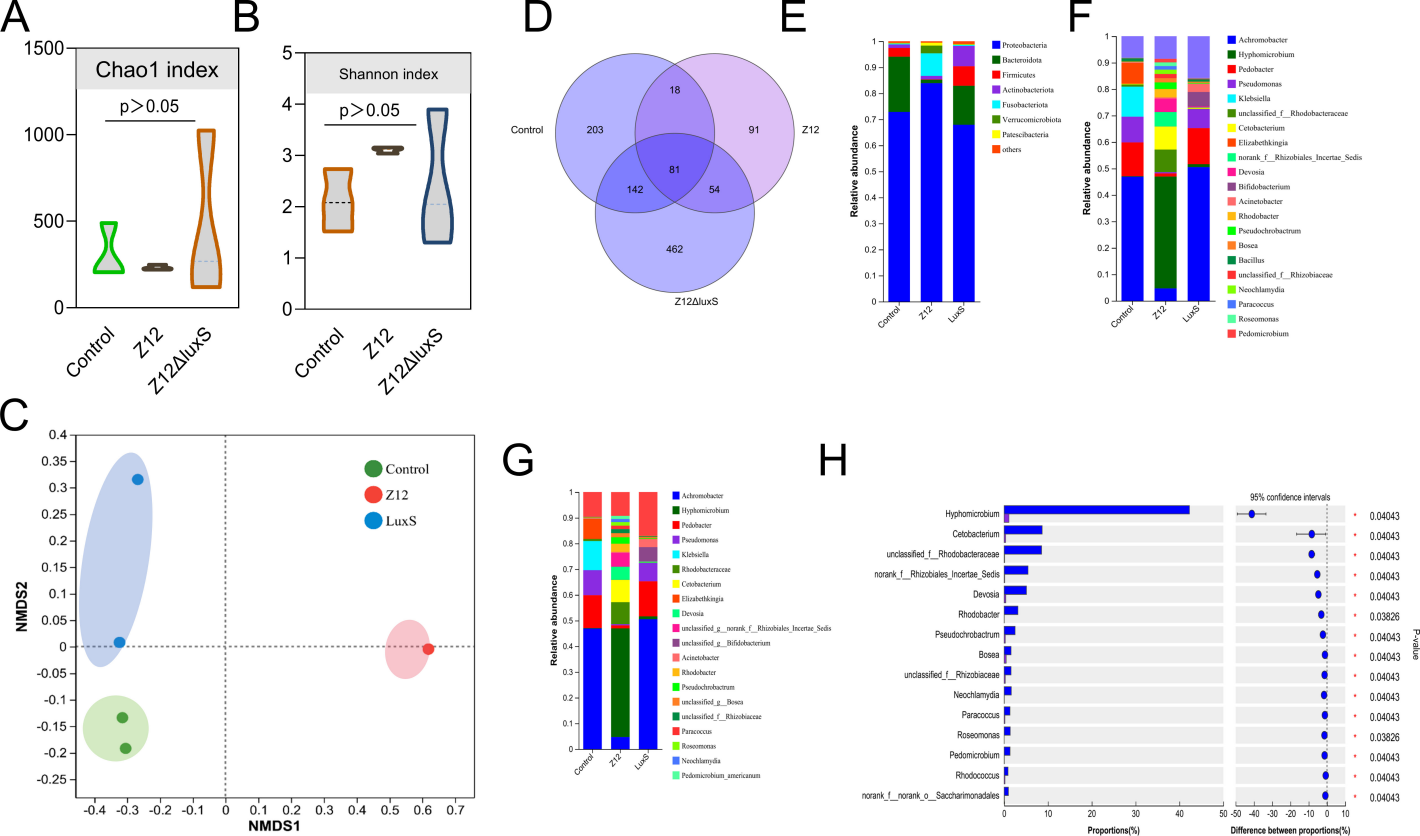
Analysis of intestinal microbiota diversity infected by different *A. veronii* strains. (**A**) Chao1 index. (**B**) Shannon index. (**C**) Beta diversity based on NMDS analysis. (**D**) OTU distribution of Venn diagram. (**E**) Species composition at the phylum level. (**F**) Species composition at the family level. (**G**) Species composition at the genus level. (**H**) Differences in species composition between Z12 and Z12Δ*luxS*.

The 16S diversity analysis revealed potential mechanisms underlying intestinal damage caused by *A. veronii* colonization. The α-diversity analysis, represented by Chao1 and Shannon indices, was higher in the Z12-treated group compared to other treatment groups, albeit without significant differences (Fig. 5A and B). β-diversity, analyzed through NMDS, showed separate clustering of Control and Z12Δ*luxS*, with significant differences in community composition within each group (Fig. 5C). While intra-group differences were reduced in Z12Δ*luxS*, overlap in the Z12-treated group indicated significant changes in the host intestinal microbiota structure upon addition of wild-type Z12, with less pronounced effects upon addition of Z12Δ*luxS*. Venn diagram analysis revealed 81 common OTUs among the three treatment groups, with Control, Z12, and Z12Δ*luxS* having 203, 91, and 462 OTUs, respectively (Fig. 5D).

Differential species were classified at the phylum, family, and genus levels. At the phylum level, Proteobacteria exhibited the highest abundance in all treatment groups, ranging from 67.9% to 83.9%. Bacteroidota ranked second in abundance in Control and Z12Δ*luxS*, at 21.2% and 14.9%, respectively, with a significant decrease in Z12-treated group, while Fusobacteriota significantly increased, ranking second at 8.6% in the Z12 group (Fig. 5E). At the family level, *Alcaligenaceae* had the highest abundance in Control and Z12Δ*luxS*, at 46.9% and 50.5%, respectively, with a significant decrease in the Z12-treated group. *Hyphomicrobiaceae* showed a significant increase, reaching 43.6%, with *Rhodobacteraceae* ranking second at 13.6% in the Z12 group (Fig. 5F). At the genus level, *Achromobacter* had the highest abundance in Control and Z12Δ*luxS*, at 46.8% and 50.5%, respectively, with a significant decrease in the Z12-treated group to 4.6%. *Hyphomicrobium* showed a significant increase, reaching 42.2%, with *Pedobacter* ranking second at 1.8% in the Z12 group (Fig. 5G). Comparison between Z12 and Z12Δ*luxS* groups revealed significant differences in abundance, particularly in *Hyphomicrobium*, followed by *Cetobacterium*, *Rhodobacteraceae*, *Rhizobiales*, and *Devosia* (Fig. 5H). Accordingly, *A. veronii* colonization significantly alters the structure of the host intestinal microbiota, resulting in significant increases and decreases in the abundance of *Hyphomicrobium* and *Achromobacter*, respectively.

### Intestinal microbiota dysbiosis induced by *A. veronii* colonization is closely related to intestinal cell apoptosis

**Fig 6:**
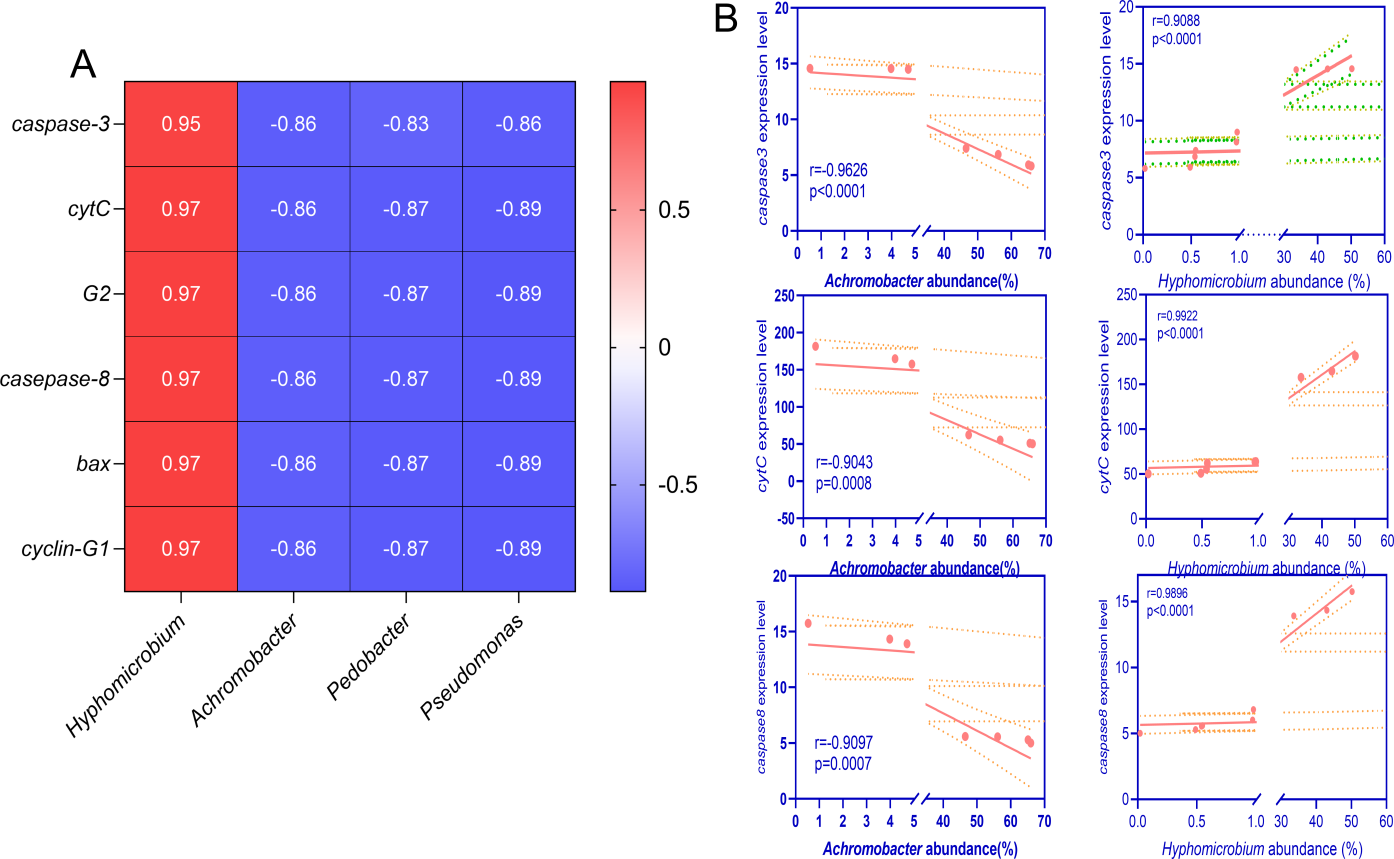
Correlation analysis between p53 pathway-related genes expression and intestinal microbiota. (**A**) Correlation analysis of *Hyphomicrobium*, *Achromobacter*, *Pedobacter*, and *Pseudomonas* with p53-related genes expression. (**B**) Analysis of the correlation between the abundance of the genera *Achromobacter* and *Hyphomicrobium* and the expression levels of *caspase-3*, *cytochrome c* (*cytC*), and *caspase-8*.

To further investigate the relationship between intestinal microbiota and host cell apoptosis, we performed Spearman correlation analyses between selected bacterial genera showing significant differences in abundance across treatment groups and p53-related genes. As depicted in Fig. 6A, the selected bacterial genera exhibited high correlations with p53 pathway-related genes. Except for a positive correlation between the abundance of *Hyphomicrobium* and p53 pathway-related genes, the other three bacterial genera showed negative correlations with p53 pathway gene expression.

Comparing the abundance changes between the Z12 and Z12Δ*luxS* groups, the bacterial genera showing the greatest increase (*Hyphomicrobium*) and decrease (*Achromobacter*) were analyzed for their correlation with key genes of the p53 pathway, including *caspase-3*, *cytC*, and *caspase-8*. The results revealed a significantly positive correlation between the expression levels of these genes and the abundance change of *Hyphomicrobium*, with correlation coefficients (*r*-values) of 0.9088 for *caspase-3*, 0.9922 for *cytC*, and 0.9896 for *caspase-8*. Conversely, a remarkably negative correlation was observed between the abundance change of *Achromobacter* and the expression of the aforementioned genes, with *r*-values of −0.9562, −0.9043, and −0.9097, respectively (Fig. 6B). Altogether, these findings suggest that alterations in intestinal microbiota are a major contributing factor to the activation of the p53 pathway.

### The elevated ROS levels triggered by nitrite accumulation stand as the primary catalyst for inducing cell apoptosis

**Fig 7:**
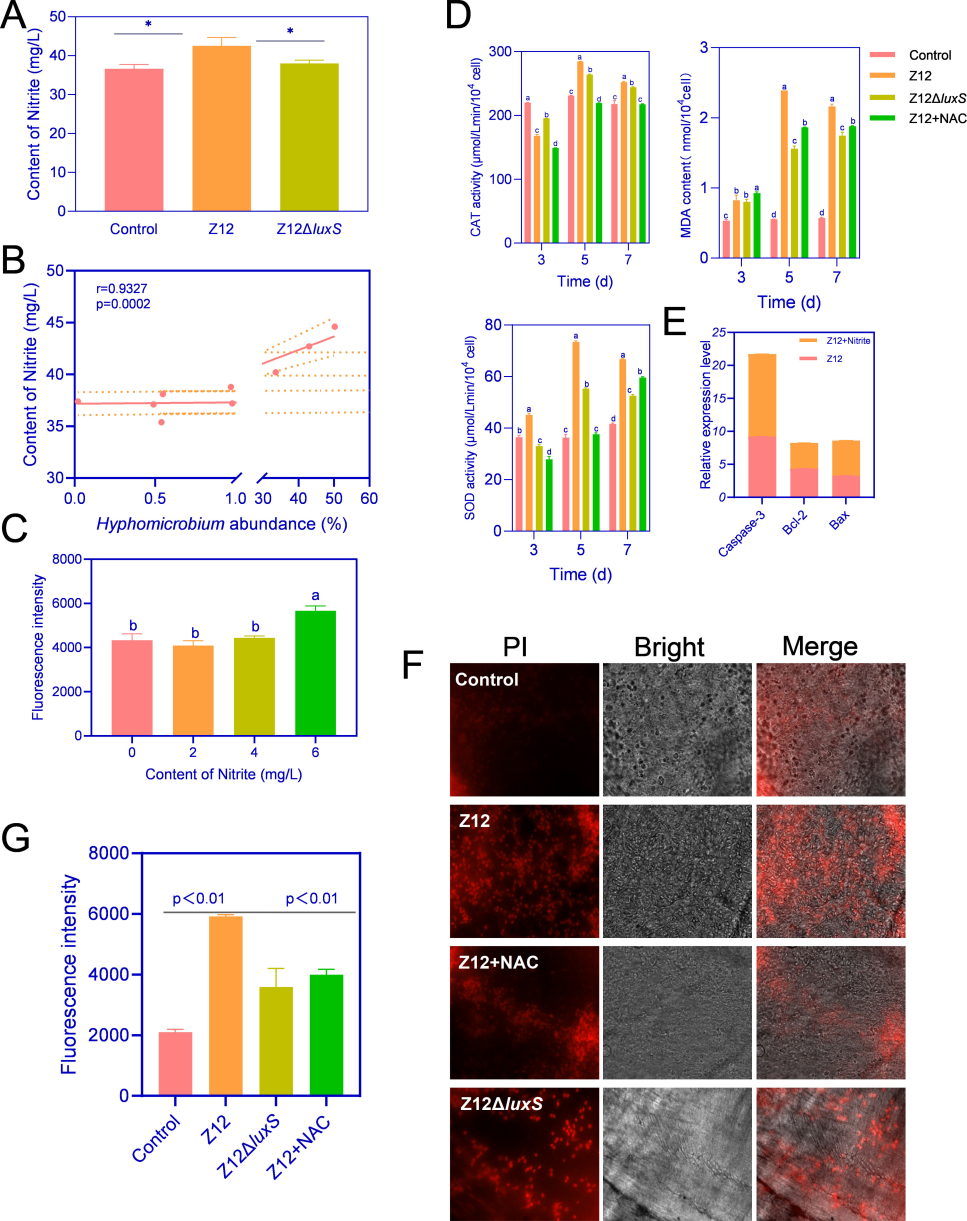
Cell apoptosis induced by elevated ROS levels with nitrite accumulation as the primary catalyst. (**A**) Nitrite content in the intestines between different treatment groups. (**B**) Correlation analysis between the nitrite content in the intestines and the abundance of *Hyphomicrobium*. (**C**) Fluorescence probe assay to detect changes in ROS levels induced by different concentrations of nitrites, with fluorescence values indicating ROS content. (**D**) Activity and levels of CAT, SOD, and MDA at different time points. (**E**) Expression levels of p53 pathway-related genes in the Z12 treatment group with and without adding nitrites. (**F, G**) Fluorescence microscopy to detect dead cells in intestinal tissues under different treatments, where dead cells were labeled by PI with red fluorescence. * indicates a statistically significant difference of *P* < 0.05. Means of groups not sharing a common letter are significantly different at *P* < 0.05.

Combining 16S diversity analysis, we observed a significant increase in the abundance of *Hyphomicrobium* in the Z12 group, which is a typical denitrifying bacterium capable of producing and accumulating nitrites. Consequently, we measured the nitrite levels in the intestines of different treatment groups, revealing a significantly higher nitrite content in the Z12 group compared to the Control and Z12Δ*luxS* groups (Fig. 7A), showing a strong positive correlation with the abundance of *Hyphomicrobium* (Fig. 7B). Furthermore, we assessed the intestinal reactive oxygen species (ROS) levels after treatment with different concentrations of nitrites. The results, as shown in Fig. 7C, indicated that nitrite concentrations of 0–4 mg/L did not significantly alter ROS levels in the intestines, whereas 6 mg/L of nitrites activated ROS levels in the host intestines.

This trend was consistent with our tracking observations during treatment, where we observed a gradual increase in the activities of superoxide dismutase (SOD) and catalase (CAT) in all treatment groups over time, with the Z12 treatment group showing the largest change. Addition of N-acetylcysteine (NAC), a ROS inhibitor, significantly inhibited the activities of CAT and SOD (Fig. 7D). CAT activity in the Z12Δ*luxS* treatment group was significantly lower than that in the Z12 treatment group on all days except the third day. Additionally, the malondialdehyde (MDA) content was highest in the Z12 group, followed by the Z12 + NAC group, Z12Δ*luxS* group, and Control group (Fig. 7D), similar to the trend observed in ROS levels. This suggests that the gradual accumulation of nitrites can significantly increase intestinal oxidative stress levels. Furthermore, we investigated the expression of p53 pathway genes and apoptosis under higher oxidative stress levels, revealing a significant upregulation of *caspase-3* and *bax* expression, along with a downregulation of *bcl-2* expression (Fig. 7E), which indicated that higher oxidative stress levels can activate the p53 pathway. Additionally, fluorescence microscopy observations showed that the number of propidium iodide (PI)-labeled cells was minimal in the Control group, significantly higher in the Z12 group compared to other treatment groups, and significantly decreased after the addition of NAC, similar to the Z12Δ*luxS* group, but still significantly higher than the blank control group (Fig. 7F and G).

Altogether, these results suggest that AI-2 mediated *A. veronii* colonization leads to the accumulation of nitrites in the intestines, resulting in elevated intestinal oxidative stress levels. ROS accumulation further activates the cell death program mediated by the p53 pathway, causing cell damage.

## DISCUSSION

Bacterial colonization in the host intestine is pivotal for pathogen infection (11), with quorum sensing systems serving as vital regulatory mechanisms for bacterial colonization and virulence factors. Building upon prior research, we delve deeper into the effects of AI-2 signaling on *A. veronii* colonization in the intestine and its induced intestinal microbiota dysbiosis and cell apoptosis.

In previous studies, we have confirmed that the deficiency of AHLs signaling could inhibit *A. veronii* colonization in the intestine and attenuate its damage to the host intestine (10). Here, we found that AI-2 signaling indeed has a similar effect, aligning with numerous previous studies. However, unlike what was expected, we did not observe a significant difference in the adhesion ability of the *luxS*-deficient mutant in the absence or presence of AI-2 in our experiment. This may suggest that the adhesion capability of *A. veronii* is minimally affected in the absence of AI-2 signaling. It is also possible that the exogenously added AI-2 does not significantly enhance the LuxS/AI-2 system in the mutant strain, resulting in no apparent difference in adhesion ability. Han et al. (12) also demonstrated that the deletion of the *luxS* gene in avian pathogenic *Escherichia coli* could reduce its colonization in the host liver, while Ruddell et al. (13) observed a significant decrease in its colonization ability in the chicken intestine after deletion of the *luxS* gene (13). Additionally, as an inhibitor of AI-2 signaling, D-ribose showed pronounced inhibitory effects on its intestinal colonization. The mechanism by which D-ribose inhibits AI-2 signaling is thought to involve competitive inhibition, where D-ribose competes with AI-2 molecules for binding to their receptors, potentially interfering with the synthesis or detection processes of AI-2, thus reducing its generation or signaling (14). Similarly, studies by Lee et al. (15) showed that D-ribose effectively inhibited the biofilm formation of *Streptococcus* and *Staphylococcus aureus* (15). Likewise, Liu et al. (16) noted a significant inhibitory effect of D-ribose on the biofilm of *Lactobacillus plantarum* L-ZS9, with inhibition positively correlated with D-ribose concentration (16). However, over time in our study, the inhibitory effect of D-ribose gradually diminished, leading to an increased abundance of *A. veronii* in the intestine. This may be attributed to the continuous consumption of D-ribose, resulting in a gradual decrease in concentration. Altogether, these results indicate that AI-2 signaling promotes *A. veronii* colonization in the intestine, while D-ribose serves as an effective inhibitor of AI-2 signaling, offering a preventive measure to reduce *A. veronii* damage to aquatic animals.

Intestinal health is paramount for the overall well-being of the host (17). A wealth of research indicates that invasion by pathogenic bacteria can inflict severe damage on intestinal health. For instance, we have observed that when zebrafish suffered from extensive intestinal injuries, their survival was gravely threatened, potentially leading to mortality (10). Wang et al. (18) have illustrated significant epithelial damage in the intestines of red crucian carp after infection by *Aeromonas hydrophila*, encompassing cell shedding and structural disruption, thereby significantly impacting the host’ s health (18). Similarly, our findings reveal that *A. veronii* colonization also induces substantial intestinal damage, posing a serious threat to host health. Nevertheless, further exploration is warranted to elucidate the precise mechanisms through which *A. veronii* inflicts intestinal damage.

Intestinal transcriptome analysis was performed to uncover the mechanism by which *A. veronii* triggers intestinal damage in the host. We found that *A. veronii* colonization significantly upregulates the expression levels of host cell p53 pathway-related genes, and TUNEL staining validation reveals severe apoptotic phenomena in host cells. Cell apoptosis serves both as a defense response to bacterial infection and can also cause substantial damage to host tissues (19). Following bacterial infection, host cells can induce apoptosis through various pathways, with p53 playing a crucial role as a transcription factor in responding to cellular damage. When the damage is severe or incomplete repair occurs, activation of p53 within cells can mediate cell cycle arrest and apoptosis by modulating other apoptotic factors (20). Previous studies have confirmed the phenomenon of pathogenic bacteria invasion leading to host cell apoptosis. Zhang et al. (21) have reported that grass carp infected by *A. hydrophila* can upregulate the expression of proteins such as Caspase-3 and Bcl-2 in its cells, which are important regulatory factors in the p53 apoptotic pathway (21). Additionally, studies by Dubytska and Thune (22) demonstrated that infection with *Edwardsiella ictaluri* in catfish induces host cell apoptosis via the T3SS system (22). Guo et al. (23) showed that infection of sea cucumber coelomic cavities with *Vibrio splendidus* triggers host cell apoptosis via regulation by the BAG family member BAG2 (23). Furthermore, these studies have consistently found that severe tissue damage often accompanies cell apoptosis, suggesting that the intestinal damage caused by *A. veronii* colonization in our study may be due to p53 pathway-mediated cell apoptosis.

Cell apoptosis can be caused by various factors, and based on previous researches, we speculate that intestinal microbiota dysbiosis induced by *A. veronii* colonization may be a significant contributor to cell apoptosis. 16S diversity analysis revealed that *A. veronii* colonization significantly altered the bacterial community structure in the Z12 treatment group, while the community structure in the Z12Δ*luxS* group resembled that of the control group. In the Z12 treatment group, there was a significant increase in the abundance of the phylum Firmicutes, which includes many bacterial species that can respond to AI-2 (24). The host intestinal microbiota dysbiosis induced by *A. veronii* colonization was closely related to AI-2. As an interspecific signaling molecule, AI-2 can be recognized by other intestinal bacteria that can produce AI-2, affecting the abundance of multiple phylogenetic types and altering the overall structure of intestinal microbial communities. Our findings are consistent with the study of Thompson et al. (24), which suggests that AI-2 can promote an increase in the phylum Firmicutes while reducing Bacteroidetes (24). This may be because there are more species in Firmicutes that can produce and respond to AI-2, collectively manipulating changes in the intestinal microbiota through quorum sensing. Meanwhile, changes in the abundance of these bacteria may alter the concentration of related metabolites in the intestine, thereby exacerbating the impact on the intestinal microbiota. Additionally, there was a significant increase in the abundance of *Hyphomicrobium* in the Z12 treatment group. *Hyphomicrobium* is the primary participants in the denitrification system in the environment, possessing robust denitrification capabilities (25). The denitrification process generates large amounts of nitrite, a significant water environmental pollutant with toxic effects on aquatic organisms, leading to hemoglobin denaturation and decreased oxygen transport capacity. However, there is currently no direct evidence that *Hyphomicrobium* induces cell apoptosis through nitrite accumulation via denitrification. Furthermore, combined analysis of 16S diversity and transcriptome sequencing revealed a significant correlation between changes in intestinal microbiota structure and the expression levels of p53 pathway-related genes, which suggested that intestinal microbiota dysbiosis may be a primary cause of cell apoptosis, and we hypothesize that nitrite may be a significant triggering factor.

Further research revealed that the abundance of the genus *Hyphomicrobium* in the Z12 treatment group significantly increased, leading to a substantial accumulation of nitrite and causing changes in ROS levels, inducing the expression of genes in the p53 apoptosis pathway. To some extent, the accumulation of ROS can activate the p53 pathway in host cells, resulting in cell apoptosis. Niwa-Kawakita et al. (26) found that in animals, as ROS levels increased, the expression of p53 was upregulated, and identified the ROS sensor PML as a regulator of the p53 pathway (26). Zheng et al. (27) found that oxidative stress in the process of culturing cardiomyocytes induced cell apoptosis in a dose-dependent manner when different concentrations of hydrogen peroxide were added (27). Furthermore, the study found that the activation of the p53 pathway by high levels of ROS was a significant cause of cell apoptosis and that deletion of *caspase-3* and *bax* eliminated cell apoptosis caused by oxidative stress (28). These findings are consistent with our conclusions, indicating that ROS can regulate the activity of the p53 pathway. The elevation of ROS levels leads to upregulation of the p53 pathway, suggesting that *A. veronii* colonization in the host intestine induces oxidative damage, resulting in increased ROS levels and activation of the p53 pathway.

The reasons for the elevation of intracellular ROS levels are also worth investigating. We found that as the most significantly increased genus in the Z12 group, *Nitrosomonas* possesses potent denitrification capabilities, leading to the accumulation of nitrite. Nitrite is one of the crucial environmental pollutants affecting the immune system of aquatic organisms. High concentrations of nitrite can penetrate through the gill epithelial cells and accumulate in tissues such as plasma, gills, brain, and liver, causing severe oxidative damage (29). Gao et al. (30) exposed fish to different concentrations of nitrite and observed that high concentrations of nitrite led to an increase in host intracellular antioxidant enzyme activity, indicating an increase in intracellular oxidative stress levels (30), which is consistent with our findings. In addition to causing an increase in host intracellular ROS levels, nitrite also induces cell damage and apoptosis through other pathways. Studies have characterized that exposure of shrimp to high concentrations of nitrite leads to a significant increase in the apoptosis rate, and exposure of mud crabs to different concentrations of nitrite results in varying degrees of apoptosis, with the apoptosis rate increasing with nitrite concentration (31, 32). Furthermore, many studies have demonstrated the critical role of the p53 pathway and Caspase-3 in nitrite-induced cell apoptosis. Cheng et al. (33) demonstrated that exposure to nitrite induces excessive ROS production in the host, leading to lipid peroxidation and DNA damage (33). Nitrite-induced oxidative stress activates the JNK pathway, upregulating the expression of p53 pathway-related genes and caspase-3 genes, resulting in host cell apoptosis. This indicates that the accumulation of nitrite leads to an elevation in host intracellular ROS levels, further activating the p53 pathway and promoting cell apoptosis through other potential pathways such as the JNK pathway.

In summary, our study unveils the significant impact of AI-2 signaling on *A. veronii* intestinal colonization, as well as its induced intestinal microbiota dysbiosis and cell apoptosis. We found that AI-2 signaling promotes *A. veronii* colonization, while D-ribose effectively inhibits this process, offering potential therapeutic avenues. Transcriptomic analysis revealed the molecular basis of intestinal damage, highlighting the upregulation of p53 pathway genes associated with apoptosis. Moreover, intestinal microbiota dysbiosis induced by *A. veronii* colonization associates with host cell apoptosis, leading to nitrite accumulation, which contributes to increasing ROS levels, and activation of the p53 pathway. These findings underscore the intricate interplay between pathogens, intestinal microbiota, and host immune responses in pathogen-induced intestinal damage. Future researches should be performed to explore targeted interventions to modulate quorum sensing mechanisms and intestinal microbiota for preserving intestinal health and combating bacterial infections.

## MATERIALS AND METHODS

### Bacterial strains and experimental animals

*A*. *veronii* Z12 (MN922947) and *A. veronii* Z12Δ*luxS* were cultured in LB medium at 30°C. *V. harveyi* BB170 was cultured in AB medium at 30°C, which was used as a reporter strain to determine AI-2 contents.

Loaches (*Misgurnus anguillicaudatus*) as the experimental animals, were purchased from the Freshwater Aquaculture Institute of Henan Province (Xinxiang, China). Individuals with body lengths of 8–12 cm were selected and underwent a week-long acclimation period under controlled conditions (28°C in a recirculating system with a light cycle of light/dark = 14 h/10 h).

### Determination of the adhesion and colonization ability

Bacterial cells of strain Z12, Z12 with the addition of 100 mM D-ribose, Z12Δ*luxS*, and Z12Δ*luxS* with the addition of 30% exogenous AI-2 (130 µM) (34) were harvested by centrifugation at 10,000 rpm for 10 min when its optical density at 600 nm (OD_600_) was 1.0. The harvested bacterial cells were labeled by 1 mg/mL of FITC (Sigma, USA) for 30 min, then rinsed with phosphate-buffered saline (PBS; 1.36-g KH_2_PO_4_, 0.1 mol/L NaOH, in 200p-mL distilled water, pH = 7.4) solution for three times to remove excess FITC. After which, the FITC-labeled bacterial cells were gavaged to the loach, respectively. Following the treatment for 1, 3, and 5 h, intestinal tissues were dissected from loaches in each group after anesthetized with tricaine methanesulfonate (MS-222) (100 mg/L), which were further observed and analyzed by ImageJ (v1.8.0.112).

Meanwhile, the feces of loaches in each group were collected at 2, 4, 6, 8, 10, 12, and 14 d, which were used to extract the total fecal DNA that can be employed for the analysis of the *torC* gene copies to evaluate the colonization level of *A. veronii* in the intestines, according to our previous research (3).

### Histopathological analysis

Bacterial cells of strain Z12 and Z12Δ*luxS* were harvested by centrifugation at 10,000 rpm for 10 min when OD_600_ was 1.0. Loaches in the control were fed continuously on a basal diet, while those in the Z12 and Z12Δ*luxS* groups were fed with basal diets containing bacterial cells (5.3 × 10^7^ CFU/ml) of strain Z12 and Z12Δ*luxS*. Following the treatment for 14 days, intestinal tissues were dissected from loaches in each group after anesthetized with MS-222 (100 mg/L), which were further used to perform histopathological analysis through hematoxylin (H&E) staining. The slices were observed by microscope inspection (Nikon, Japan), and analyzed based on Image Pro Plus 6.0.

### Transcriptome sequencing and 16S rRNA gene sequencing

Bacterial cells (5.3 × 10^7^ CFU/ml) of strain Z12 and Z12Δ*luxS* were gavaged to the loaches for 14 days. Then, the intestines of these loaches were dissected and rinsed with PBS for three times, which were further frozen in liquid nitrogen for 30 min. The intestinal tissues were used to perform transcriptome sequencing, meanwhile, the intestinal contents were collected for the analysis of 16S rRNA gene sequencing. Loaches in the control group were fed continuously on a basal diet and dissected to collect intestinal tissues and intestinal contents as a control.

Total RNA was extracted from intestinal tissues using Trizol regent (Macklin, China) and the genomic DNA was removed with DNase I. Library construction was performed based on the removal of rRNA using an rRNA removal kit. RNA fragments were then randomly broken into 200- to 300-bp fragments, using mRNA as a template with random oligonucleotides as primers to synthesize the first strand of cDNA. Then, the first strand was used as a template to synthesize the second cDNA strand, purified double-stranded cDNA, and amplified to obtain a specific library. Transcriptome sequencing was conducted on the Illumina NovaSeq X Plus platform. Adapters, poly-N, and low-quality sequences in the raw sequences data were removed. The assembled transcriptome unigenes were annotated based on the databases such as GO and KEGG. The DEGs were analyzed by DESeq2 (v1.38.0) using *p*-adjust < 0.05 & |log_2_FC| ≥ 1. GO enrichment analysis of DEGs was performed using GOAtools (v0.6.5), KEGG pathway enrichment analysis was analyzed based on the R package cluster-Profiler (v3.18.1).

The bacterial DNA of the intestinal contents in each group was extracted as templates to amplify the V3-V4 regions of the 16S rRNA gene. The PCR products were purified and sequenced based on an Illumina MiSeq platform (Illumina, USA). The optimized sequences were further processed to obtain the amplicon sequence variants, which were further used to perform α-diversity, β-diversity, and community composition analysis.

### Quantitative real-time PCR

The total RNA of intestinal tissues was extracted based on the RaPure Total RNA Plus Kit (Megea, China), which was used to generate cDNA based on M5 HiperScript II Reverse Transcriptase (Mei5 Biotechnology, China). The qRT-PCR was performed using a fluorescence quantitative PCR (Roche, Switzerland) based on 2 × SYBR premix WizTaq I (Nobelab, China). The primer pairs in this study for qRT-PCR are listed in Table S1, with GAPDH as a reference gene. The relative gene expression levels were quantified using the 2^−ΔΔCt^ method.

### Immunofluorescence detection

Meanwhile, the intestines of loaches in the above groups (Control, Z12, and Z12Δ*luxS*) were fixed in paraformaldehyde for 24 h and dehydrated, which were further embedded in paraffin for sectioning, then the paraffin sections were dewaxed with xylene and hydrated with alcohol, followed by incubation with proteinase K at 37°C for 30 min. Then, they were rinsed three times with PBS, and then terminal deoxynucleotidyl transferase enzyme and fluorescent label were added and incubated in the dark at 37°C for 1 h. Subsequently, 50 µL of TUNEL detection liquid was added and incubated in the dark at 37°C for 6 min. Then, 2-(4-amidinophenyl)-6-indolecarbamidine dihydrochloride (DAPI) was added and incubated at room temperature in the dark for 30 min, then washed, and determined by confocal laser scanning microscopy imaging (Leica TCS SP8, Germany) using an excitation wavelength of 488 nm for green fluorescence (TUNEL signal) and 405 nm for blue fluorescence (DAPI), with emission captured at 520 and 460 nm, respectively.

For determination of immunofluorescence, the intestines in each group were dissected, which were further fixed in paraformaldehyde at room temperature and rinsed with PBS. Subsequently, the intestinal tissues were treated with 0.5% Triton-X100 for 20 min, and PBS containing 10% goat serum was added for blocking for 30 min. After which, the goat serum was discarded, and the diluted primary antibody was added for overnight incubation at 4°C. After rinsing with PBS for three times, the fluorescence-labeled secondary antibody (1:300) was added and incubated at 37°C for 1 h in the dark, rinsed with PBS three times, and then DAPI staining solution was added and incubated at room temperature in the dark for 10 min, rinsed three times with PBS, and observed under a fluorescence microscope (Olympus BX53, Japan) using an excitation wavelength of 550 nm for red fluorescence (secondary antibody signal) and 405 nm for DAPI, with emissions captured at 580 and 460 nm, respectively. Images were acquired using a 40× objective lens with a numerical aperture of 0.75. Exposure times and gain settings were kept consistent across all groups to ensure comparability.

### Determination of intestinal oxidative stress level and nitrite concentration

Loaches in the control group were fed continuously on a basal diet, while those in the treatment groups were fed with basal diets containing bacterial cells (5.3 × 10^7^ CFU/ml) of strain Z12, Z12Δ*luxS*, and strain Z12 with the addition of NAC (Sigma, USA), respectively. After which, intestinal tissues were dissected from loaches in each group and homogenized. Subsequently, the supernatant in the homogenate was harvested by centrifugation at 12,000 rpm for 10 min, which was used to measure the activities of CAT, SOD, and the MDA content based on the specific assay kits (Grace Biotech, China).

The intestines were dissected from loaches in each group and rinsed with PBS for three times, and then, dichlorodihydrofluorescein diacetate (Beyotime, China) as a fluorescent probe was added and incubated at room temperature in the dark for 15 min. After which, the fluorescent-labeled intestines were homogenized, and the supernatant was harvested to measure the fluorescent value through a multi-mode microplate detection system (PerkinElme, USA).

The intestines were dissected from loaches in each group and rinsed with sterile water for three times to collect the intestinal contents, which were used to measure the nitrite content in it based on the nitrite content detection reagent kit (J&L Biological, China).

### Statistical analysis

All data were presented as mean ± standard deviation. For comparisons between two groups, independent samples *t*-tests were applied. For multiple group comparisons, one-way analysis of variance (ANOVA) followed by Tukey’ s post hoc test was conducted for normally distributed data, while the Kruskal–Wallis test with Dunn’ s post hoc test was used for nonparametric data. PCA was conducted using R software (v4.3.0) with the relevant packages. Statistical significance was defined as a *P* < 0.05.

## Data Availability

The intestinal transcriptome raw reads were deposited in the National Center for Biotechnology Information (NCBI) database under accession numbers SRR28961214, SRR28961215, and SRR28961216. The 16S rRNA raw reads were deposited in the NCBI database under accession numbers SRR28956758, SRR28956759, and SRR28956760.

## SUPPLEMENTAL MATERIAL

Table S1 aem.00143-25-s0001.docx Primers used for qRT-PCR amplification in this study.

ASM does not own the copyrights to Supplemental Material that may be linked to, or accessed through, an article. The authors have granted ASM a non-exclusive, world-wide license to publish the Supplemental Material files. Please contact the corresponding author directly for reuse.
