# Supplementary material for: Autoinducer-2 signaling promotes intestinal colonization of Aeromonas veronii and induces cell apoptosis in loach (Misgurnus anguillicaudatus)
Source: Appl Environ Microbiol. 2025 Feb 13;91(3):e00143-25. doi: 10.1128/aem.00143-25 (PMC11921386; doi:10.1128/aem.00143-25)
Supplement: Table S1 — Primers used for qRT-PCR amplification in this study. [file aem.00143-25-s0001.docx]

**SUPPLEMENTAL MATERIAL**

**Autoinducer-2** **signaling promotes intestinal colonization of** ***Aeromonas veronii* and induces** **cell apoptosis in loach** **(*Misgurnus anguillicaudatus*)**

Yi Li^a, b^, Shuo Han^a, b^, Wenfang Niu^a, b^, Chao Gao^a, b^, Yuqi Wang^a, b^, Mengyuan Qin^a, b^, Jingjing Han^c^, Xiaohua Xia^a^, Hailei Wang^a, b *^

^a^ College of Life Sciences, Henan Normal University, Xinxiang 453007, China

^b^ Henan Province Engineering Laboratory for Bioconversion Technology of Functional Microbes, Xinxiang 453007, China

^c^ School of Medicine, Qingdao Huanghai University, Qingdao 266299, China

Table S1 Primers used for qRT-PCR amplification in this study.

| Gene | Primers sequences |
| --- | --- |
| GAPDH | F: 5’-ACTGTTGACGGACCCTCTGGAA-3’ |
|  | R: 5’-ACGGGAACACGGAAAGCCAC-3’ |
| *caspase-3* | F: 5’-TCTTCAGAGGGGACTGTTGC-3’ |
| *bax*  *bcl-xL*  *sival*  *cytC*  *cdc-2* | R: 5’-GCCAGGAAAAGTAACCAGGTG-3’  F: 5’-CATGGGCTGGACATTGGAC-3’  R: 5’-CACTTCAGCGACTCAGCCAA-3’  F: 5’-ATGACCGAGTACCTGAACCG-3’  R: 5’-TCCACAAAGGCGTCGGAT-3’  F: 5’-GAACCTCACCAGCATGTCCA-3’  R: 5’-TGCCAAGGTGTCCGCATTAT-3’  F: 5’-AAAGGGAGCAAGCACAAGACTG-3’  R: 5’-TCCAGGTACGCCATCAGTGTCTC-3’  F: 5’-GATGATTGCCATCTCCTTTC-3’  R: 5’-AAAGCTTCAGCCAGTTGTTCA-3’ |
